# Interactions of Notch1 and TLR4 signaling pathways in DRG neurons of *in vivo* and *in vitro* models of diabetic neuropathy

**DOI:** 10.1038/s41598-017-15053-w

**Published:** 2017-11-02

**Authors:** Tianhua Chen, Hao Li, Yiting Yin, Yuanpin Zhang, Zhen Liu, Huaxiang Liu

**Affiliations:** 1 Department of Rheumatology, Shandong University Qilu Hospital, Jinan, 250012 China; 2 Department of Orthopaedics, Shandong University Qilu Hospital, Jinan, 250012 China; 3 Department of Anatomy, Shandong University School of Basic Medical Sciences, Jinan, 250012 China

## Abstract

Understanding the interactions between Notch1 and toll-like receptor 4 (TLR4) signaling pathways in the development of diabetic peripheral neuropathy may lead to interpretation of the mechanisms and novel approaches for preventing diabetic neuropathic pain. In the present study, the interactions between Notch1 and TLR4 signaling pathways were investigated by using dorsal root ganglion (DRG) from diabetic neuropathic pain rats and cultured DRG neurons under high glucose challenge. The results showed that high glucose induced not only Notch1 mRNA, HES1 mRNA, and TLR4 mRNA expression, but also Notch1 intracellular domain (NICD1) and TLR4 protein expression in DRG neurons. The proportion of NICD1-immunoreactive (IR) and TLR4-IR neurons in DRG cultures was also increased after high glucose challenge. The above alterations could be partially reversed by inhibition of either Notch1 or TLR4 signaling pathway. Inhibition of either Notch1 or TLR4 signaling pathway could improve mechanical allodynia and thermal hyperalgesia thresholds. Inhibition of Notch1 or TLR4 signaling also decreased tumor necrosis factor-α (TNF-α) levels in DRG from diabetic neuropathic rats. These data imply that the interaction between Notch1 and TLR4 signaling pathways is one of the important mechanisms in the development or progression of diabetic neuropathy.

## Introduction

A wide array of cell signaling mediators and their interactions play vital roles in neuroinflammation associated neurodegeneration^1^. Toll-like receptors (TLRs) may regulate the processes of neurogenesis and neurite outgrowth, suggesting their roles in neuronal plasticity^2^. Increasing evidence suggests that Toll-like receptor 4 (TLR4) contributes importantly to chronic pain sensitization^3^. The role of TLR4 in diabetes mellitus has been receiving much attention at present. TLR4-mediated chronic inflammation not only causes many diabetes complications such as diabetic neuropathy, but also has a profound impact on the internal environment of the body and microenvironment of the nervous system^4^. Since TLR4 is widely distributed in the nervous system and also has an important role in the regulation of neuroinflammation, the unique role of TLR4 in diabetic neuropathy should be further clarified.

Inflammatory responses could play a critical role in the pathogenesis of neuron injury^5^. Tumor necrosis factor-α (TNF-α) is a downstream pro-inflammatory cytokine of TLR4 signaling pathway^6,7^. Activation of TLR4 induces production or release TNF-α in DRG^8^. Increased expression of pro-inflammatory cytokines such as TNF-α in the peripheral nervous system suggests the possibility of change in pain perception in diabetes^9,10^. The possibility exists that TLR4 signaling is correlated with the modulation of inflammatory mediator TNF-α and increase the sensitivity of nociception.

The Notch signaling pathway is crucial for regulation of neuronal differentiation and survival^11,12^. Notch signaling regulates the fate of cells in the developing nervous system^13,14^ and is important in synaptic plasticity and inflammation in the nervous system^15^. Defects in the expression of Notch genes result in severe, often lethal, development abnormalities^16^. Notch1 receptor plays a role in modulation of synaptic activity of distinct growth factor, which shows insights into a possible cytokine/Notch signaling cross-talk complex^17^. Chronic pain associated with chronic neuroinflammation is caused by a local inflammation in the peripheral nervous system. Both TLR4 and Notch signaling contributed to the induction and maintenance of mechanical allodynia in neuropathic pain^8,15^. TLR4 and Notch signaling may be crucial in the progression of painful diabetic neuropathy. However, their interactions in the process of initiating and developing diabetic neuropathy remain elusive. On the track of the crucial role for Notch1 and TLR4 signaling pathways in regulation of diabetic neuropathy, this study aims to investigate the involvement of specific interactions of Notch1 and TLR4 signaling pathways by using both streptozotocin (STZ)-induced diabetic rat model *in vivo* and cultured DRG neurons under high glucose challenge *in vitro*.

N-[N-(3,5-difluorophenacetyl)-1-alanyl]-S-Phenylglycinet-butylester (DAPT) is served as an inhibitor for Notch1 receptor through decreasing the expression of Notch intracellular domain (NICD) as well as the downstream gene hairy and enhancer of split 1 (HES1)^18^. DAPT has potent and specific characteristics of inhibition of γ-secretase, which specifically blocks the Notch1 pathway by preventing the intracellular domain of Notch1 (NICD1) from being released and translocated to the nucleus. The decrease of NICD1 expression was detected after DAPT treatment^19^. Whether the inhibitory effect of DAPT on NICD1 expression prevents the progression of diabetic neuropathy and relieves diabetic neuropathic pain should be further evaluated. TAK-242 (TAK) is a novel antagonist of TLR4 and effectively decreases neuroinflammation by its efficacy on blocking TLR4 to bind with its downstream adaptor protein^1,20^. In many experimental conditions, inhibition of TLR4 by TAK induced decreases of TLR4 expression^1,20,21^. The effect of TAK on reducing neuroinflammation in diabetic neuropathy should be further explored. In the present study, both DAPT and TAK were used as the specific inhibitors for detecting the interactions of Notch1 and TLR4 in the progression of diabetic neuropathy. Especially, the interactions of Notch1 and TLR4 signaling were focused in this study for their influence on reliving pain behavior. The interactions of different signaling pathways might provide a novel anti-neuroinflammatory strategy with additional therapeutic options for developing diabetic neuropathy.

## Results

### The alterations of mechanical and thermal thresholds after inhibition of Notch1 or TLR4

**Figure 1:**
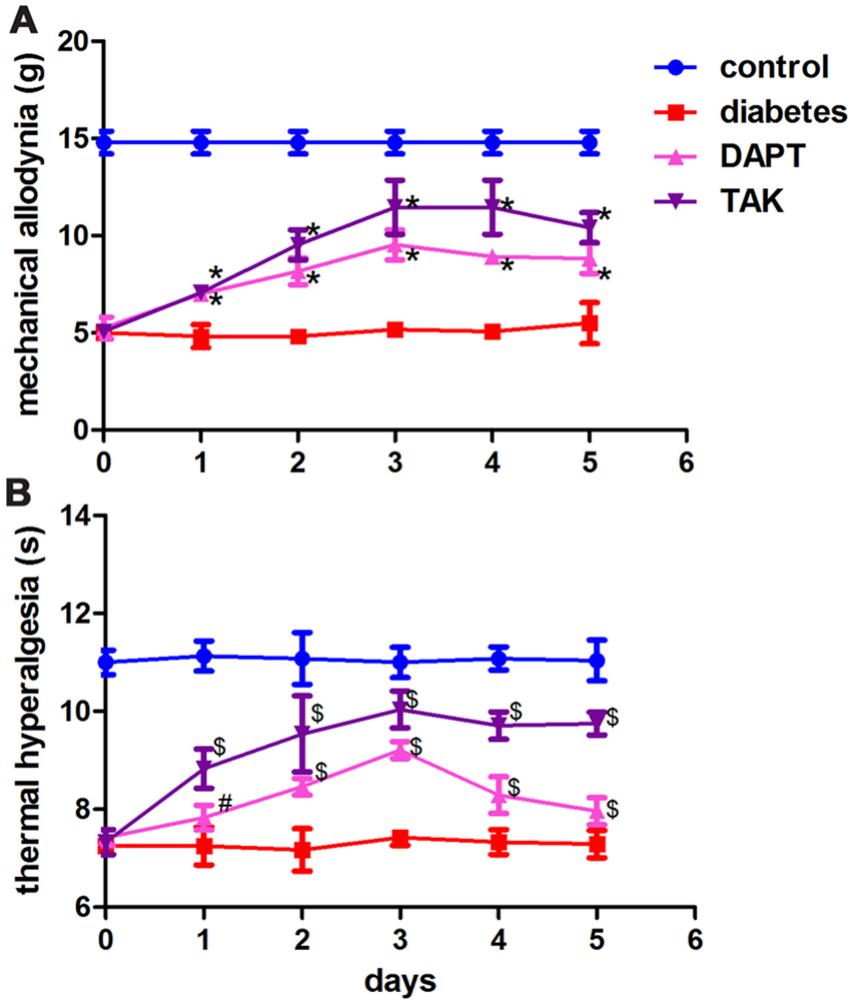
Neuropathic pain behavior test with different treatment. (**A**) The mechanical thresholds. Bar graphs with error bars represent mean ± SD. *P < 0.001. (**B**) The thermal thresholds. Bar graphs with error bars represent mean ± SD. ^#^ *P* < 0.01, ^$^ *P* < 0.001.

According to the convergent evidence that activation of TLR4 contributes the development of neuropathic pain and its possible interactions with Notch1 signaling, inhibition of either Notch1 or TLR4 signaling on mechanical allodynia and thermal hyperalgesia of painful diabetic neuropathic rats was tested in this study. After adminstration of STZ for 6 weeks, the rats were intrathecally injected the Notch1 inhibitor DAPT (10 μl, 5 mg/ml) or the TLR4 inhibitor TAK (10 μl, 10 mg/ml), respectively, once per day for 3 consecutive days. The mechanical and thermal thresholds were measured once per day for 5 consecutive days after first injection of DAPT or TAK. Inhibition of either Notch1 or TLR4 elevated the mechanical and thermal thresholds, which reflected the improvement of the neuropathic pain behaviors. The highest point of mechanical and thermal thresholds was at 3 days after injection of Notch1 inhibitor DAPT or TLR4 inhibitor TAK. The mechanical and thermal thresholds were also maintained at a high level in 2 days after stopping injection compared with the painful diabetic neuropathic animals without injection of Notch1 inhibitor DAPT or TLR4 inhibitor TAK. These data imply that inhibition of either Notch1 or TLR4 signaling pathway might be a novel therapeutic target for relieving pain behaviors of diabetic neuropathy (Fig. 1).

### The mRNA levels of Notch1, HES1, and TLR4 in DRG *in vivo* and *vitro*

**Figure 2:**
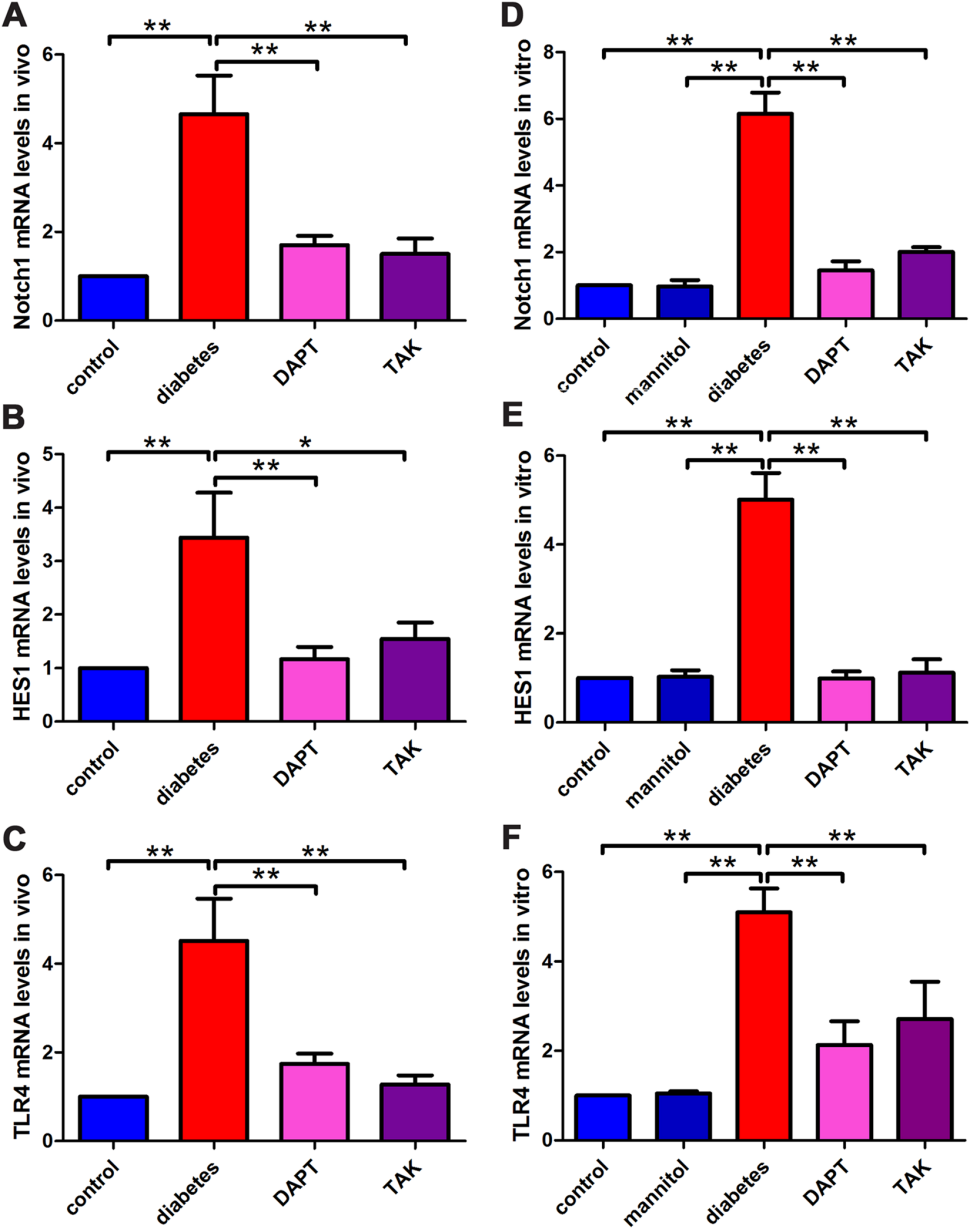
The mRNA levels of Notch1, HES1, and TLR4 in DRG neurons *in vivo* and *in vitro*. (**A**) Notch1 mRNA levels in DRG neurons from painful diabetic neuropathic rats. (**B**) HES1 mRNA levels in DRG neurons of from painful diabetic neuropathic rats. (**C**) TLR4 mRNA levels in DRG neurons of from painful diabetic neuropathic rats. (**D**) Notch1 mRNA levels in cultured DRG neurons. (**E**) HES1 mRNA levels in cultured DRG neurons. (**F**) TLR4 mRNA levels in cultured DRG neurons. Bar graphs with error bars represent mean ± SD. *P < 0.01, **P < 0.001.

The mRNA levels of Notch1, HES1, and TLR4 in L4-L6 DRG from diabetic neuropathic pain rats and cultured DRG neurons under high glucose challenge were determined using real-time PCR analysis by measurement the mRNA transcription level. Activation of Notch1 downstream signaling HES1 has been shown to regulate neural differentiation^22^. HES1 mRNA expression in this study was also determined. The mRNA levels of Notch1, HES1, and TLR4 *in vivo* were shown in Table 1. The mRNA levels of Notch1, HES1, and TLR4 *in vitro* were shown in Table 2. The result showed that the mRNA levels of Notch1, HES1, and TLR4 elevated in DRG neurons from STZ-induced painful diabetic neuropathic rats and cultured DRG neurons under high glucose challenge. After inhibition of either Notch1 or TLR4 signaling pathway, the mRNA levels of Notch1, HES1, and TLR4 decreased in DRG neurons, which suggested the interactions of the two signaling pathways (Fig. 2).

**Table 1:** Notch1, HES1, and TLR4 mRNA levels of DRG *in vivo*.

mRNA levels/control	diabetic neuropathy	DAPT	TAK
Notch1 mRNA	4.66 ± 0.87	1.70 ± 0.21**	1.50 ± 0.35**
HES1 mRNA	3.44 ± 0.84	1.17 ± 0.23**	1.55 ± 0.30*
TLR4 mRNA	4.52 ± 0.95	1.74 ± 0.23**	1.28 ± 0.20**

Compared with diabetic neuropathy group, *P < 0.01, **P < 0.001.

**Table 2:** Notch1, HES1, and TLR4 mRNA levels of DRG *in vitro*.

mRNA levels/control	mannitol	high glucose	DAPT	TAK
Notch1 mRNA	0.97 ± 0.19**	6.15 ± 0.64	1.45 ± 0.27**	2.00 ± 0.15**
HES1 mRNA	1.03 ± 0.15**	5.01 ± 0.60	0.99 ± 0.16**	1.12 ± 0.30**
TLR4 mRNA	1.05 ± 0.05**	5.10 ± 0.53	2.13 ± 0.53**	2.71 ± 0.84**

Compared with high glucose group, **P < 0.001.

### The protein levels of NICD1 and TLR4 in DRG *in vivo* and* in vitro*

**Figure 3:**
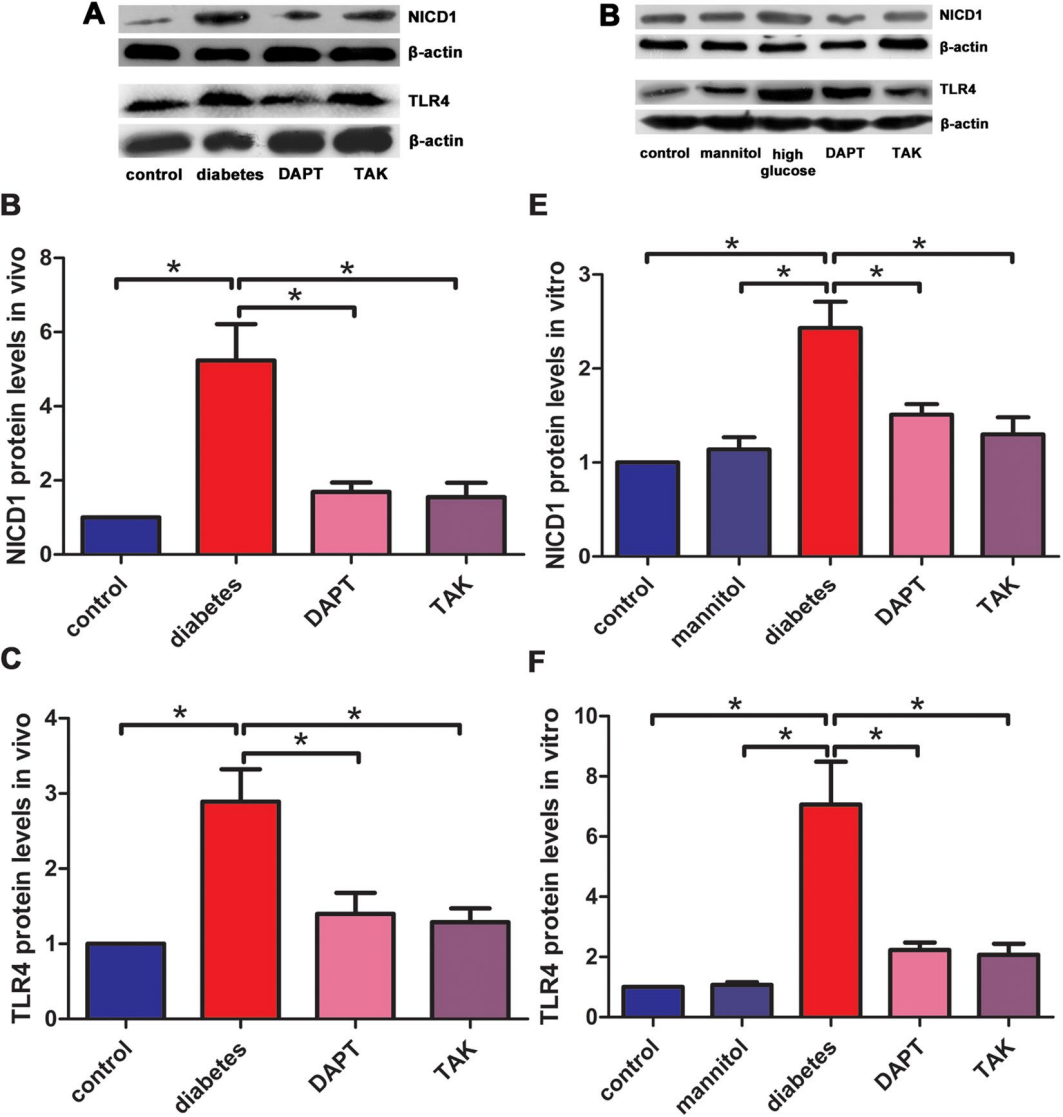
The protein levels of NICD1 and TLR4 in DRG *in vivo* and vitro. (**A**) Immunoblotting bands of NICD1 and TLR4 in DRG from painful diabetic neuropathic rats. (**B**) Quantification of NICD1 protein levels in DRG from painful diabetic neuropathic rats. (**C**) Quantification of TLR4 protein levels in DRG from painful diabetic neuropathic rats. (**D**) Immunoblotting bands of NICD1 and TLR4 in cultured DRG neurons. (**E**) Quantification of NICD1 protein levels in cultured DRG neurons. (**F**) Quantification of TLR4 protein levels in cultured DRG neurons. Bar graphs with error bars represent mean ± SD. *P < 0.001.

The protein levels of NICD1 and TLR4 in L4-L6 DRG from diabetic neuropathic pain rats and cultured DRG neurons under high glucose challenge were determined by Western blot assay. The protein levels of NICD1 and TLR4 *in vivo* were shown in Table 3. The protein levels of NICD1 and TLR4 *in vitro* were shown in Table 4. The protein levels of NICD1 and TLR4 elevated in DRG neurons from STZ-induced painful diabetic neuropathic rats and cultured DRG neurons under high glucose challenge. After inhibition of either Notch1 or TLR4 signaling pathway, the protein levels of NICD1 and TLR4 decreased in DRG neurons. These results suggested that high glucose challenge could induce activation both Notch1 and TLR4 signaling pathways. After inhibition of either Notch1 or TLR4 signaling pathway, both Notch1 and TLR4 signaling pathways were affected, which suggested the interactions of the two signaling pathways. This might be one of the mechanisms of high glucose induced peripheral diabetic neuropathy or neurotoxicity (Fig. 3).

**Table 3:** NICD1 and TLR4 protein levels of DRG *in vivo*.

protein levels/control	diabetic neuropathy	DAPT	TAK
NICD1	5.24 ± 0.98	1.69 ± 0.25*	1.55 ± 0.39*
TLR4	2.89 ± 0.43	1.40 ± 0.28*	1.29 ± 0.18*

Compared with diabetic neuropathy group, *P < 0.001.

**Table 4:** NICD1 and TLR4 protein levels of DRG *in vitro*.

protein levels/control	mannitol	high glucose	DAPT	TAK
NICD1	1.14 ± 0.13*	2.43 ± 0.28	1.51 ± 0.11*	1.30 ± 0.18*
TLR4	1.07 ± 0.09*	7.06 ± 1.43	2.23 ± 0.25*	2.07 ± 0.37*

Compared with high glucose group, *P < 0.001.

### The protein levels of TNF-α in DRG from diabetic animal model

**Figure 4:**
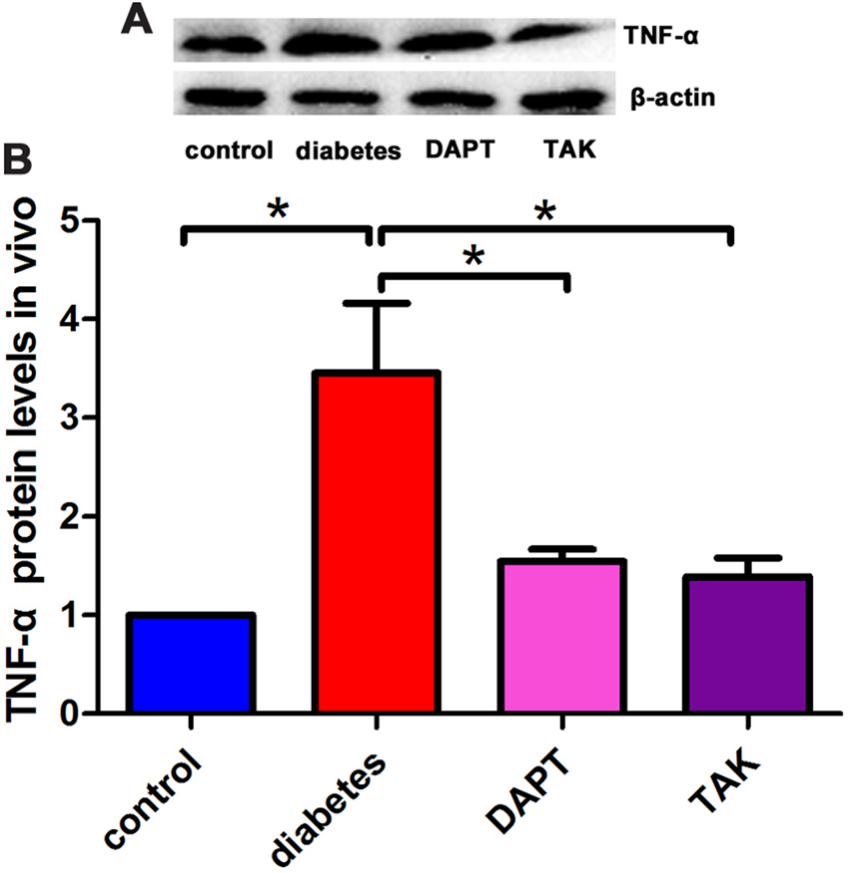
The protein levels of TNF-α in DRG from painful diabetic neuropathic rats. (**A**) Immunoblotting bands of TNF-α. (**B**) Quantification of TNF-α protein levels. Bar graphs with error bars represent mean ± SD. *P < 0.001.

TNF-α is a down stream pro-inflammatory cytokine of TLR4. In this study, the levels of TNF-α in DRG from diabetic animal model were determined by Western blot assay after inhibition of either Notch1 or TLR4 signaling pathway. The TNF-α protein level in diabetic rats, DAPT treated diabetic rats, and TAK treated diabetic rats was 3.45 ± 0.71, 1.55 ± 0.12, and 1.39 ± 0.19 folds of control, respectively. TNF-α levels were elevated in DRG from diabetic animal model. Inhibition of either Notch1 or TLR4 signaling decreased TNF-α level in DRG. This result suggested that Notch1 or TLR4 activation promoted TNF-α expression is one of the mechanisms of painful diabetic neuropathy. Inhibition of Notch1- or TLR4-TNF-α signaling pathway might be an effective target for relieving diabetic neuropathic pain behaviors (Fig. 4).

### The expression of NICD1 or TLR4 in situ of cultured DRG neurons

**Figure 5:**
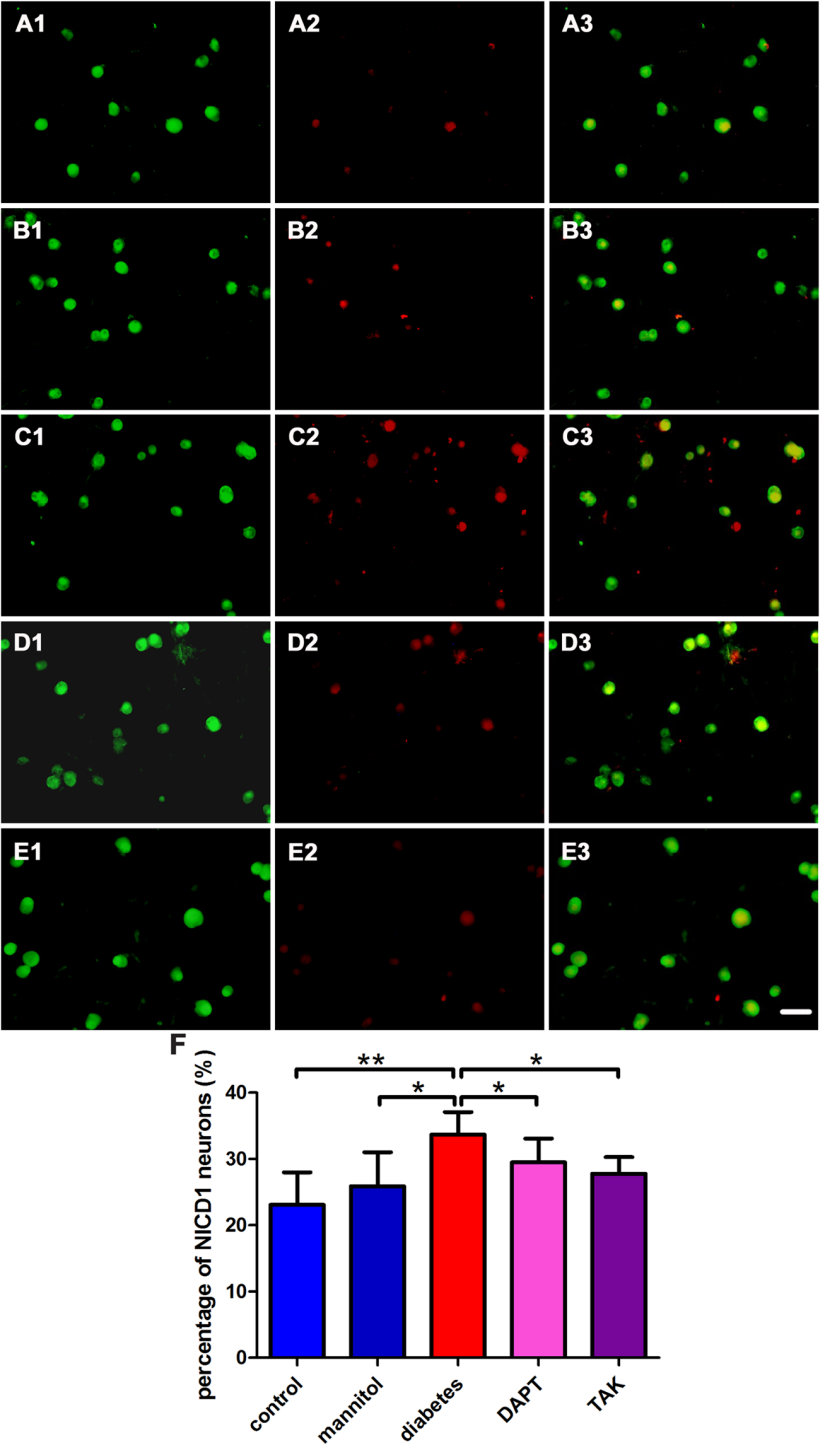
Double fluorescence labeling of MAP2 and NICD1 *in situ* of cultured DRG neurons. (**A**) (control): A1, MAP2-IR neurons; A2, NICD1-IR neurons; A3, overlay of A1 and A2. (**B**) (mannitol): B1, MAP2-IR neurons; B2, NICD1-IR neurons; B3, overlay of B1 and B2. (**C**) (high glucose): C1, MAP2-IR neurons; C2, NICD1-IR neurons; C3, overlay of C1 and C2. (**D**) (high glucose + DAPT): D1, MAP2-IR neurons; D2, NICD1-IR neurons; D3, overlay of D1 and D2. (**E**) (high glucose + TAK): E1, MAP2-IR neurons; E2, NICD1-IR neurons; E3, overlay of E1 and E2. (**F**). Quantification of the percentage of NICD1-IR neurons. Bar graphs with error bars represent mean ± SD. Scale bar = 50 μm. *P < 0.05, **P < 0.001.

**Figure 6:**
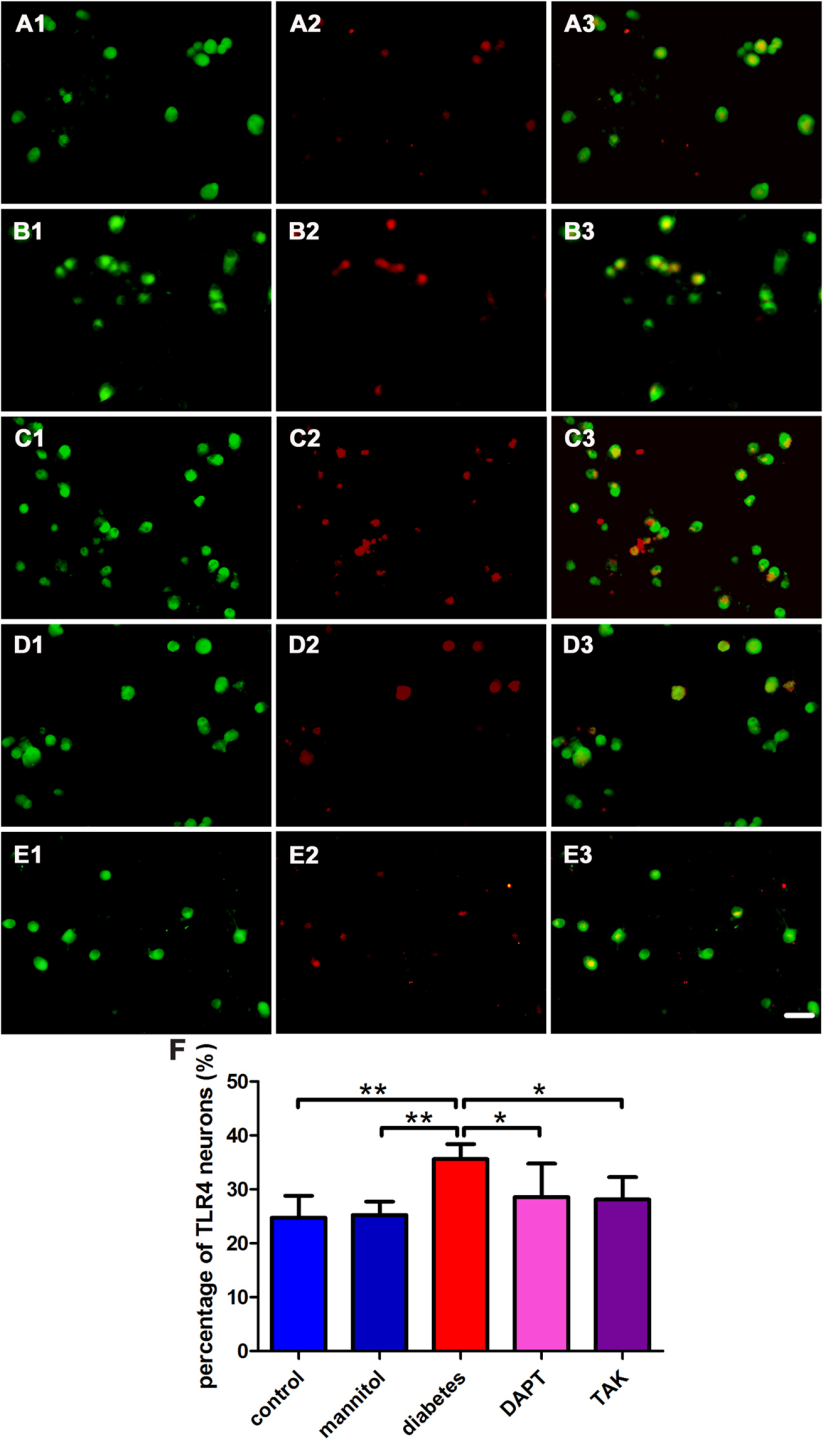
Double fluorescence labeling of MAP2 and TLR4 *in situ* of cultured DRG neurons. (**A**) (control): A1, MAP2-IR neurons; A2, TLR4-IR neurons; A3, overlay of A1 and A2. (**B**) (mannitol): B1, MAP2-IR neurons; B2, TLR4-IR neurons; B3, overlay of B1 and B2. (**C**) (high glucose): C1, MAP2-IR neurons; C2, TLR4-IR neurons; C3, overlay of C1 and C2. (**D**) (high glucose + DAPT): D1, MAP2-IR neurons; D2, TLR4-IR neurons; D3, overlay of D1 and D2. (**E**) (high glucose + TAK): E1, MAP2-IR neurons; E2, TLR4-IR neurons; E3, overlay of E1 and E2. (**F**). Quantification of the percentage of TLR4-IR neurons. Bar graphs with error bars represent mean ± SD. Scale bar = 50 μm. *P < 0.05, **P < 0.001.

The expression of NICD1 or TLR4 *in situ* in cultured DRG neurons under high glucose challenge were detected by double fluorescence labeling of microtubule-associated protein 2 (MAP2) and NICD1 or TLR4. The percentage of NICD1-immunoreactive (IR) neurons in control, mannitol, high glucose, high glucose + DAPT, high glucose + TAK group was 23.1% ± 4.9%, 25.9% ± 5.1%, 33.7% ± 3.4%, 29.5% ± 3.6%, and 27.8% ± 2.5%, respectively. The percentage of TLR4-IR neurons in the corresponding group was 24.7% ± 4.1%, 25.2% ± 2.5%, 35.6% ± 2.8%, 28.6% ± 6.2%, and 28.1% ± 4.2%, respectively. High glucose challenge increased the proportion of NICD1-positive and TLR4-positive neurons. After inhibition of either Notch1 or TLR4 signaling pathway, the proportion of NICD1-positive and TLR4-positive neurons decreased (Figs 5 and 6).

## Discussion

**Figure 7:**
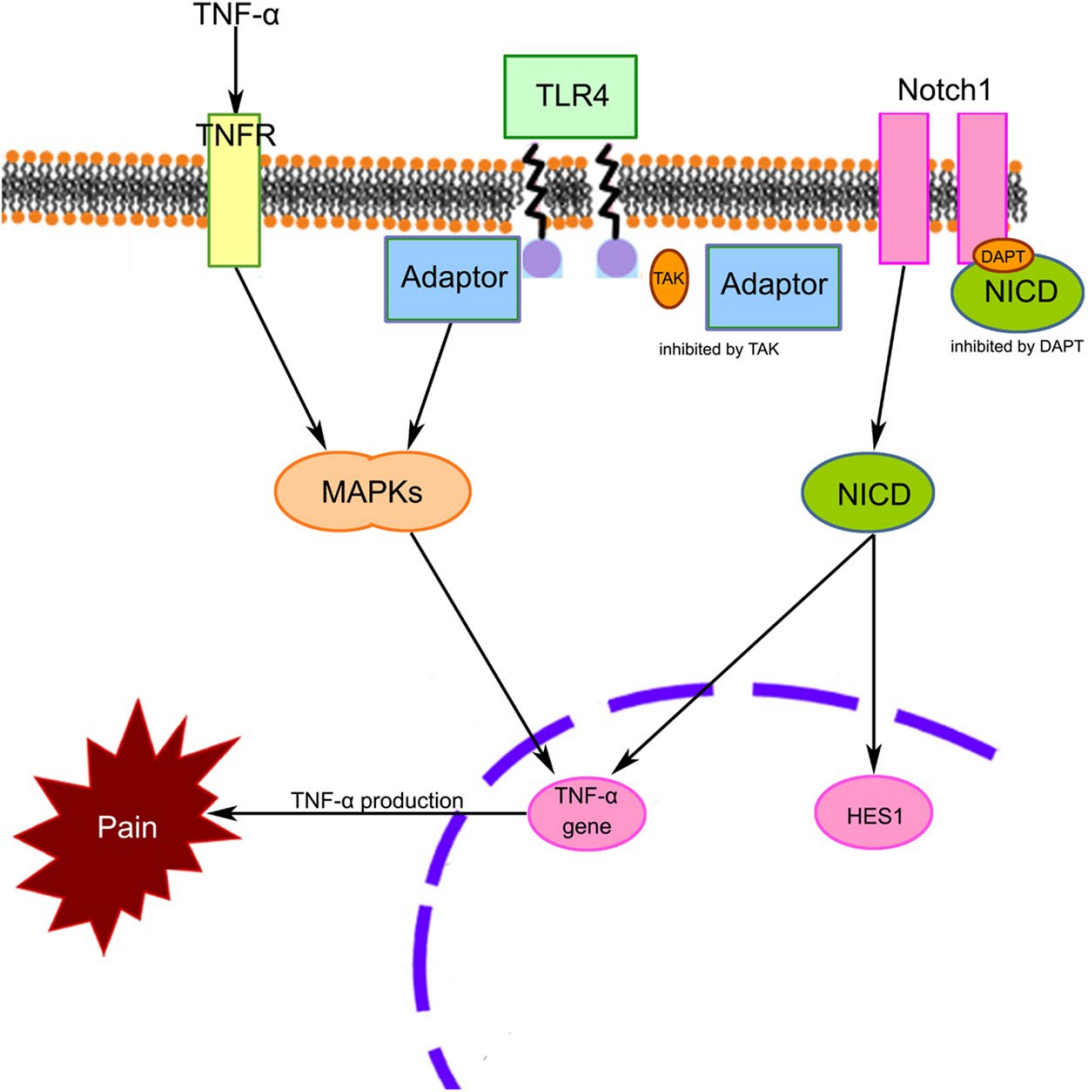
The pain related mechanisms of Notch1 and TLR4 signaling pathway. Inhibition of either Notch1 or TLR4 signaling pathway could alleviate diabetic neuropathic pain. Inhibition of Notch1 or TLR4 signaling also decreased TNF-α level in DRG from diabetic neuropathic rats.

DRG was necessary for transduction of inflammatory pain signal caused by high glucose in the diabetic neuropathy model. Diabetic neuropathic rats exhibited mechanical allodynia and thermal hyperalgesia. Intrathecal injection of either Notch1 inhibitor DAPT or TLR4 inhibitor TAK ameliorated diabetic neuropathic pain behaviors, which implied that inhibition of either Notch1 signaling or TLR4 signaling alleviated painful diabetic neuropathy. In the DRG dissected directly from diabetic neuropathic pain rats and those cultured under high glucose challenge, the mRNA expression is increased not only in Notch1 and HES1, also in TLR4. The protein levels of NICD1 and TLR4 is also elevated in the aforementioned experimental conditions. The proportion of NICD1-positive and TLR4-positive neurons in DRG cultures is also increased under high glucose challenge. Inhibition of Notch1 or TLR4 signaling also decreased TNF-α level in DRG from diabetic neuropathic rats. Summing up, inhibition of Notch1 or TLR4 signaling pathway improved primary sensory neuronal status in diabetic neuropathic pain rats and those cultured under high glucose challenge (Fig. 7).

The interesting part of the present result is that application of either one of the inhibitor could inhibit two signaling pathways. HES1, a downstream signaling transcription factor (TF) of Notch1 in neuron^20^, also showed reduction in mRNA expression with its upstream Notch1. Interestingly, interactions of Notch1 and TLR4 signaling pathway play an essential role in DRG of the diabetic neuropathy model because inhibition of one pathway could affect the activation of the other. Our findings suggest that TLR4 signaling in DRG is important for diabetic neuropathic pain that may underlie potential interactions with the Notch1 signaling pathway.

TLR4-mediated signaling pathway is associated with the promotion of neuroinflammatory response in neurons^23,24^. TLR signaling pathway mediates neuroinflammation and expression of cytokines in DRG neurons^25^. Activation of TLR4 in DRG sensory neurons could influence excitation of these neurons and contribute to neuropathic pain^26^. Several studies dealt with that the pro-inflammatory cytokine TNF-α correlates to the diabetic peripheral neuronal abnormalities^27–29^. A recent clinical trial has shown that TNF-α may contribute to the pro-inflammatory status of the distal sensory nerves in patients with diabetes or prediabetes^27^. In the present study, TNF-α was upregulated in DRG from STZ-induced diabetic neuropathic pain rats. It has been shown that plasma concentrations of inflammatory indicators including TNF-α were positively correlated with TLR4 in patients with diabetic peripheral neuropathy^30^, which may aggravate the TLR4-mediated inflammatory cascade that is already activated. The results of our present study demonstrated that induction of inflammation contributed to the maintenance of neuropathic pain, suggesting a potential therapeutic target for the treatment of neuropathic pain.

When TLR4 signaling, or inflammation, is inhibited by TAK, it not only may have affected the interactions of TLR4 signaling and with members of Notch1 signaling, but also interfered with Notch-dependent transcription. On the other hand, Notch1 inhibitor DAPT inhibited the expression of TLR4 suggesting the role of Notch1 signaling on survival of affected neurons. The fact that Notch signaling pathway is crucial for neuronal differentiation and survival suggested that interactions between TLR4 with members of Notch1 signaling regulate certain aspects of latency of the neurons affected diabetic neuropathy. Consequently, for this study, we compared the effects of the inhibitors of both signaling pathways to test whether TLR4 inhibitor TAK interfered with members of Notch1 signaling and Notch1 inhibitor DAPT with TLR4. TLR4 inhibitor TAK consistently interfered with members of Notch1 signaling molecules and TLR4 was also consistently inhibited by Notch1 inhibitor DAPT.

It is well known that the inflammation effects are involved in the pain signal transduction in the diabetic neuropathy model in addition to their damage to the axons. TLR4 signaling also promotes repair after demyelinating conditions such as spinal cord injury^31^. However, there may be conflicting roles of different signaling pathways particularly of their effects on the neuronal activity. In this study, the pain behaviors developed in the painful diabetic neuropathy model were reversed by either Notch1 inhibitor DAPT or TLR4 inhibitor TAK, suggested that different pathways were involved. However, these pathways are actually playing a competing role in neuronal survival. The involvement of the Notch1 receptor in diabetic neuropathy is pro-neuronal survival which in turn promotes neuronal activity. Notch1 inhibition may or may not have direct influence on the inhibitory effect of the inflammation induced by diabetic neuropathy, but might be due to the decreased activity of neurons related to the pain behaviors by regulating the fate of these cells.

Notch receptors are shown to regulate nociceptive thresholds^15,16^, because Notch could provide a proper balance of excitatory and inhibitory neurons of neuronal subtype specification^32^. In our present study, mechanical allodynia is developed in the diabetic neuropathy model. In this process, the excessive activation of Notch signaling contributed to the induction and maintenance of mechanical allodynia because more excitatory neurons are activated. Administering DAPT significantly prevented the nociceptive effects because the balance of excitatory and inhibitory neuronal activation is restored. These data suggested that Notch must be considered as important new targets of the diabetic neuropathy.

In conclusion, intrathecal administration of Notch1 inhibitor DAPT and TLR4 inhibitor TAK attenuated allodynia and hyperalgesia. PCR and Western blot analysis revealed time-dependent upregulation of TLR4 mRNA and protein, Notch1 mRNA, NICD1 protein, which was paralleled by pain behavior of the established diabetic neuropathy. High glucose challenge for cultured DRG neurons similarly influenced the Notch1 and TLR4 signaling pathways in agreement with diabetic animal model. Blockade of either Notch1 or TLR4 signaling pathway produced anti-nociceptive effects and inhibited the expression of TLR4 mRNA and protein, Notch1 mRNA, NICD1 protein, which suggests that they may be putative interactions when the pain of diabetic neuropathy is developed and may be the target for future pharmacological pain relief tools. Summing up, in light of the interactions of Notch1 and TLR4 signaling pathways, both Notch1 and TLR4 signaling pathway may indeed play a significant role in development of neuropathy, which could be linked to the nociceptive behavior in diabetic neuropathy.

## Materials and Methods

### Ethics Statement

Compliance with the National Institute of Health Guide for the Care and Use of Laboratory Animals (eighth edition, 2010; International Standard Book Number-13: 978-0-309-15400-0; http://www.nap.edu), all animals were carefully treated during the experiments. Especially, all surgery was performed under anesthesia and all efforts were made to minimize suffering of the animals. All procedures or protocols described here were reviewed and approved by the Ethical Committee for Animal Experimentation of Shandong University.

### Preparations of diabetic neuropathic pain animal model

All preparations utilized male rats (200 g~250 g) taken from the breeding colony of Wistar rats maintained in the Experimental Animal Center at Shandong University of China. All the rats were housed in plastic cages with a normal light-dark cycle and allowed free access to rat chow and water. Diabetic neuropathic pain rat model was induced by a single peritoneal injection (i.p.) of STZ (55 mg/kg freshly dissolved in 0.1 mol/L citric acid buffer, pH 4.5) as our previous study^33^. Seven days later, blood glucose level from the tail vein was measured by a strip-operated reflectance meter. Blood glucose level ≥20 mmol/L was recognized as hyperglycemia. Age-matched control rats received an injection of equivalent volume normal saline. All the rats showed hyperglycemia at the beginning and end of this study after STZ injection manifested with evident mechanical allodynia and thermal hyperalgesia. This is a well successfully established diabetic neuropathic pain rat model.

### Catheter implantation

For intrathecal injection (i.t.), a sterile polyethylene catheter (PE-10, 8.0 cm length) (Instech Laboratories Incorporation, Plymouth Meeting, PA) was inserted through an incision in the gap between the L4/L5 vertebrae and the tip of catheter was positioned at the subarachnoid space under anesthesia with of 3% pentobarbital sodium (1 ml/kg, i.p.). After a 3-day recovery period and at the end of the experiment, 2% lidocaine (10 μl, i.t.) was given through the catheter to make sure the catheter tip was in appropriate position. Only the rat showing reversible hind limb motor deficits immediately following the lidocaine injection were considered to be catheterized successfully.

### Animal groups

Thirty-two rats were randomly divided into 4 groups (8 rats in each group): (1) Control group: The equivalent volume normal saline (10 μl) was administrated (i.t.) for normal rat as a control. (2) Diabetic group: After STZ injection for 6 weeks, normal saline (10 μl) was administrated (i.t.) for diabetic rat once per day for 3 consecutive days. (3) DAPT group: After STZ injection for 6 weeks, the Notch1 inhibitor DAPT (10 μl, 5 mg/ml) was administrated (i.t.) for diabetic rat once per day for 3 consecutive days. (4) TAK group: After STZ injection for 6 weeks, the TLR4 inhibitor TAK (10 μl, 10 mg/ml) was administrated (i.t.) for diabetic rat once per day for 3 consecutive days. After each injection, artificial cerebrospinal fluid (ACSF, 10 μl) was used immediately for removing residual liquid in the catheter. The ACSF was composed of following ingredients (mmol/L): NaCl 138.6, KCl 3.35, CaCl_2_·2H_2_O 1.26, MgCl_2_·6H_2_O 1.16, NaH_2_PO_4_·2H_2_O 0.58, NaHCO_3_21.0, and glucose 10.0, pH was adjusted to 7.4.

### Evaluation of mechanical allodynia and thermal hyperalgesia

The mechanical allodynia and thermal hyperalgesia were assessed by determination of the withdrawal threshold by means of a von Frey filaments (BME-403, Chinese academy of medical sciences institute of biomedical engineering) and a plantar analgesia tester (BME-400C, Chinese academy of medical sciences institute of biomedical engineering), respectively. The detailed measurement of mechanical allodynia and thermal hyperalgesia was carried out as our previous study^34^. After STZ injection for 6 weeks, the threshold of mechanical allodynia and thermal hyperalgesia was measured once per day for 5 consecutive days.

### DRG cell culture preparations

The paradigm of regenerative state of cultured DRG neurons is useful for *in vitro* study^35^. Cultured DRG neurons under high glucose challenge were served as an *in vitro* model of diabetic neuropathy^36^. These culture preparations utilized newborn rats (less than 24 h after birth) taken from the breeding colony of Wistar rats maintained in the Experimental Animal Center at Shandong University of China. Dissociated DRG cell culture procedure was similar to our previous study^37^. Briefly, the DRG was digested with 0.25% trypsin (Sigma, St. Louis, MO) and centrifuged at 1 × 10^3^ rpm. The pellets were resuspended in Dulbecco’s Modified Eagle Medium with F-12 supplement (DMEM/F-12) medium (Gibco, Grand Island, NY). DRG cells for fluorescent labeling were plated at 1 × 10^5^ cells/well in 24-well clusters (Costar, Corning, NY) which would contain a coverslip precoated with poly-L-lysine (0.1 mg/ml, Sigma, St. Louis, MO) in each well. DRG cells for real-time PCR and Western blot assay were plated at a density of 5 × 10^5^ cells/ml. The composition of the culture medium is DMEM/F-12 (1:1) supplemented with 5% fetal bovine serum, 2% B-27 supplement (Gibco, Grand Island, NY), L-glutamine (0.1 mg/ml, Sigma, St. Louis, MO). Dissociated DRG cells were cultured at 37 °C with 5% CO_2_ for 24 hours and then maintained in culture medium containing cytarabine (ara-C) (5 μg/ml) for another 24 hours to inhibit growth of non-neuronal cells. After that, neurons were cultured in neurobasal medium for additional 72 hours at different experimental conditions before observation.

### Exposure of different agents to cultured DRG neurons

DRG neurons at 48 h post-culture were randomly divided into 5 groups: (1) Control group: Neurobasal medium contains 25 mmol/L of glucose which is optimal for DRG neuron survival and growth, so this condition of glucose concentration was used in control group. (2) Mannitol group: Mannitol 20 mmol/L was used here to create a high osmotic pressure mimicking the high glucose condition. (3) High glucose group: Additional 20 mmol/L glucose was added to the medium as high glucose challenge. (4) High glucose + DAPT group: Additional 20 mmol/L glucose and 10 μmol/L of DAPT were added to the neurobasal medium. (5) High glucose + TAK group: Additional 20 mmol/L glucose and 10 μmol/L of TAK were added to the neurobasal medium. The cultured DRG neurons were maintained in distinct conditions for 72 hours before observation. Each condition was performed in triplicate as one experiment. Eight experiments (n = 8) were used for final analysis.

### Real-time PCR analysis for Notch1 mRNA, HES1 mRNA, and TLR4 mRNA levels

The L4-L6 DRG from each animal after behavioral tests and the cultured DRG neurons in distinct conditions were processed for real-time PCR analysis for detecting Notch1 mRNA, HES1 mRNA, and TLR4 mRNA levels. The procedures of real-time PCR analysis were similar to our previous study^34^. The synthetic oligonucleotide primer sequences for Notch1, HES1, TLR4, and β-actin were as follows: Notch1 5′-GTG AGT GGG ATG GAC TGG AC-3′ (coding sense), 5′-GGA AGG AGT TGT TGC GTA GC-3′ (coding antisense); HES1 5′-TCG GTG GTT ACT TTG TTG CT-3′ (coding sense), 5′-AGG CGC AAT CCA ATA TGA AC-3′ (coding antisense); TLR4 5′-TGG CAT CAT CTT CAT TGT CC-3′ (coding sense), 5′-CAG AGC ATT GTC CTC CCA CT-3′ (coding antisense); β-actin 5′-CAC CCG CGA GTA CAA CCT TC-3′ (coding sense), 5′-CCC ATA CCC ACC ATC ACA CC-3′ (coding antisense). The final results of real time-PCR were expressed as the ratio of mRNA of control.

### Western blot assay for NICD1, TLR4, and TNF-α protein levels

TLR4 and NICD1 protein levels in DRG from diabetic neuropathic pain animals and cultured DRG neurons under high glucose challenge were detected by Western blot assay. TNF-α protein levels in DRG from diabetic neuropathic pain animals were also detected by Western blot assay after Notch1 inhibitor DAPT or TLR4 inhibitor TAK treatment. The detailed Western blot assay procedures were as described in our previous study^34^. The first antibodies used in this study were as follows: mouse anti-NICD1 monoclonal IgG (1:500, Abcam, HK), mouse anti-TLR4 monoclonal IgG (1:500, Abcam, HK), rabbit anti-TNF-α polyclonal IgG (1:1,000, Abcam, HK), and mouse anti-β-actin monoclonal IgG (1:1,000, Abcam, HK). The second antibodies were as follows: goat anti-rabbit IgG-HRP (1:3,000, Santa Cruz Biotechnology, Santa Cruz, CA) and goat anti-mouse IgG-HRP (1:3,000, Santa Cruz Biotechnology, Santa Cruz, CA). The relative levels of TLR4, NICD1, and TNF-α were normalized to control.

### Double fluorescence labeling of MAP2 and NICD1 or TLR4 of cultured DRG neurons

Cultured DRG neurons under different conditions were processed for double fluorescence labeling of MAP2 and NICD1 or TLR4. The double fluorescence labeling procedures were similar to our previous study^34^. The first antibodies were mouse anti-NICD1 monoclonal IgG (1:500, Abcam, HK), mouse anti-TLR4 monoclonal IgG (1:500, Abcam, HK), mouse and chicken anti-MAP2 monoclonal IgG (1:500, Abcam, HK). The second antibodies were goat anti-mouse conjugated to Cy2 (1:100, Abcam, Cambridge, UK) and goat anti-chicken conjugated to Cy3 (1:200, Abcam, Cambridge, UK). Upon finishing fluorescence labeling, NICD1-IR and TLR4-IR neurons were counted in five visual fields of each coverslip. One visual field was in the center of the coverslip. The other four visual fields were just adjacent to, but not overlap with, the central visual field at the upper, lower, left, and right side, respectively. The MAP2-IR neurons in the same visual field were also counted as the total number of neurons. Then the percentage of NICD1-IR and TLR4-IR neurons could be obtained.

### Statistical analysis

All experiments were performed in triplicate for each condition as one experiment. Eight experiments (n = 8) were finished for final analysis. All the results were reported as mean ± SD. Non-parametric test was used to compare the abnormally distributed variables. ANOVA was used to compare the normally distributed variables. Post hoc multiple comparative analysis method was used after ANOVA had tested out that the values among various groups are different. If the normally distributed variable is homogeneous of variance, Student-Newman-Keuls test was used. If the normally distributed variable is heterogeneous of variance, Dunnett’s T3 test. All these tests were analyzed by using SPSS (version 19.0) software with *P* value < 0.05 used to delineate significance for analysis of all results.
